# Differential roles for the adapters Gads and LAT in platelet activation by GPVI and CLEC-2

**DOI:** 10.1111/j.1538-7836.2008.03166.x

**Published:** 2008-12

**Authors:** C E HUGHES, J M AUGER, J McGLADE, J A EBLE, A C PEARCE, S P WATSON

**Affiliations:** * Centre for Cardiovascular Sciences, Institute of Biomedical Research, Medical School, University of BirminghamBirmingham, UK; † Department of Medical Biophysics, Hospital for Sick ChildrenToronto, ON, Canada; ‡ Center for Molecular Medicine, Department of Vascular Matrix Biology, Frankfurt University HospitalFrankfurt, Germany

**Keywords:** CLEC-2, Gads, GPVI, LAT, platelet, signalosome, SLP-76

## Abstract

*Background:*The adapter proteins SLP-76 and LAT have been shown to play critical roles in the activation of PLCγ2 in platelets downstream of GPVI/FcRγ and the C-type lectin receptor CLEC-2. SLP-76 is constitutively associated with the adapter Gads in platelets, which also binds to tyrosine phosphorylated LAT, thereby providing a potential pathway of regulation of SLP-76. *Objective:*In the present study, we have compared the role of Gads alongside that of LAT following activation of the major platelet glycoprotein receptors using mice deficient in the two adapter proteins. *Results:*Gads was found to be required for the efficient onset of aggregation and secretion in response to submaximal stimulation of GPVI and CLEC-2, but to be dispensable for activation following stronger stimulation of the two receptors. Gads was also dispensable for spreading induced through integrin α_IIb_β_3_ or the GPIb–IX–V complex. Further, Gads plays a negligible role in aggregate formation on collagen at an arteriolar rate of shear. In stark contrast, platelets deficient in the adapter LAT exhibit a marked decrease in aggregation and secretion following activation of GPVI and CLEC-2, and are unable to form stable aggregates on collagen at arteriolar shear. *Conclusions:*The results demonstrate that Gads plays a key role in linking the adapter LAT to SLP-76 in response to weak activation of GPVI and CLEC-2 whereas LAT is required for full activation over a wider range of agonist concentrations. These results reveal the presence of a Gads-independent pathway of platelet activation downstream of LAT.

## Introduction

Immunoreceptor-tyrosine-based-activation motifs (ITAMs) are characterized by the presence of two YxxL/I motifs separated by 6–12 amino acids and are present on the cytosolic chains of a number of hematopoietic-specific immunoglobulin receptors, including Fc receptors, T- and B-cell antigen receptors (TCR and BCR), and the platelet collagen receptor complex, GPVI–FcRγ chain. This group of receptors signals through Src family kinase-dependent phosphorylation of the conserved tyrosines in the ITAM leading to recruitment of a Syk family kinase via its tandem SH2 domains, and stimulation of a downstream signaling cascade, which results in activation of phospholipase C (PLC) γ. A novel variation of this signaling pathway has been described in which the C-type lectin family receptors, CLEC-2, Dectin-1, and CLEC-9A, signal through sequential activation of Src and Syk family tyrosine kinases downstream of a single YxxL sequence [1–3].

Adapter proteins play a critical role in signaling by ITAMs and the ITAM-like receptors described above by forming a protein-scaffold that serves to recruit PLCγ to the membrane. This is illustrated by the role of the membrane adapter LAT and the two cytosolic adapters, Gads (Mona/Grap2/GrpL/Grf40) and SLP-76 in regulation of PLCγ1 in T cells. Gads binds constitutively to SLP-76 and associates with LAT upon T-cell receptor (TCR) activation forming a LAT signalosome that is critical for activation of PLCγ1 [4]. Mice deficient in LAT and SLP-76 have a complete inhibition of pre-TCR signaling, leading to an absence of circulating, mature T cells [5,6]. Similarly, there is a complete loss of activation of PLCγ1 by the TCR in LAT-deficient or SLP-76-deficient Jurkat cells [7,8]. In contrast, there is a limited degree of signaling by the pre-TCR in the absence of Gads, resulting in a reduced number of mature T cells [9]. Furthermore, disruption of the interaction between SLP-76 and Gads has been shown to impair TCR signaling in Jurkat cells by approximately 50% [10,11]. Thus, there is an absolute requirement for LAT and SLP-76 in the activation of PLCγ1 by the TCR, whereas the role for Gads can be partially bypassed.

The LAT signalosome plays an important role in the activation of platelets by the platelet collagen receptor GPVI and the C-type lectin receptor, CLEC-2. However, in contrast to signaling through the TCR, a limited degree of activation of PLCγ2 is induced by both receptors in platelets deficient in LAT [2,12]. Furthermore, CLEC-2 is able to induce a partial activation of PLCγ2 in the absence of SLP-76, whereas this adapter is critical for the activation of the phospholipase by GPVI [2,13]. Thus, there are important differences in the roles of LAT and SLP-76 in signaling between GPVI and CLEC-2 in platelets, and also with the TCR. Interestingly, SLP-76 is required for activation of PLCγ2 downstream of two other major platelet glycoprotein receptors, integrin α_IIb_β_3_ and GPIb–IX–V [14–16]. However, in both cases, it is controversial as to whether this is mediated downstream of an ITAM-containing protein.

Very little is known about the role of Gads in platelets. Gads associates with SLP-76 in platelets and undergoes tyrosine phosphorylation upon stimulation of GPVI by the synthetic collagen, CRP [17]. In the only functional study using Gads-deficient platelets, Judd *et al.* [15] reported that Gads is dispensable for α-granule secretion in platelets induced by a high concentration of GPVI-specific agonist, convulxin, whereas LAT is essential for this response. In contrast, the same research group has shown that mutation of the Gads binding site on SLP-76 impairs GPVI-induced platelet secretion [18]. To definitively characterize the role of Gads in platelets, we have carried out an extensive investigation of Gads-deficient platelets following activation by GPVI, CLEC-2, integrin α_IIb_β_3_, and GPIb–IX–V.

## Experimental procedures

### Animals

Genetically modified mice deficient in Gads [9] and LAT [5] were bred as heterozygotes to generate knockouts and littermate controls. LAT-deficient mice were bred on a C57Bl6J background. Gads-deficient mice were bred on a Balb-c background or backcrossed for seven generations onto a C57Bl6J background.

### Reagents

Rhodocytin was purified from *Calloselasma rhodostoma* venom as previously described [19]. The α-phosphotyrosine mAb 4G10, α-SLP-76 pAb, α-Gads pAb, and α-LAT pAb were from Upstate Biotechnology Inc. (TCS Biologicals Ltd, Bucks, UK). The α-Grb2 pAb was from Santa Cruz Biotechnology (Heidelberg, Germany). All other reagents were from previously described sources [20–23].

### Platelet preparation

Venous blood from healthy drug-free volunteers was taken into 10% sodium citrate. Mouse blood was drawn from CO_2_ asphyxiated mice following isofluorane anesthesia by portal vein puncture and taken into 100 μL of acid citrate dextrose. Washed human and mouse platelets were obtained by centrifugation using prostacyclin to prevent activation during the isolation procedure [24]. Both sets of washed platelets were resuspended in modified Tyrodes buffer as previously described [24]. Platelets were used at a cell density of 2 × 10^8^ mL^−1^ for aggregometry, 5 × 10^8^ mL^−1^ for biochemical studies and 2 × 10^7^ mL^−1^ for spreading assays.

### Platelet aggregation and adenosine triphosphate secretion

Aggregation was monitored by light transmission using a Born lumi-aggregometer (Chronolog, Havertown, PA, USA). Adenosine triphosphate (ATP) secretion was measured by the addition of a luciferin/luciferase substrate/enzyme mix (Chronolume) [24,25].

### Flow adhesion studies

Mouse blood was drawn into sodium heparin (10 U mL^−1^) and PPACK (40 μm). Glass capillary tubes (Camlab, Cambridge, UK) were coated in the presence of 100 μg mL^−1^ Horm collagen (Nycomed, Munich, Germany) for 1 h at room temperature. The capillaries were washed and blocked with phosphate buffered saline (PBS) containing 5 mg mL^−1^ of heat-inactivated bovine serum albumin (BSA) for 1 h at room temperature before being mounted on the stage of an inverted microscope (DM IRB; Leica). Anticoagulated whole blood was pre-incubated with 2 μm DiOC_6_ for 15 min at 37 °C to fluorescently label the cells. It was then perfused through the chamber for 4 min at a wall shear rate of 1000 s^−1^ at 37 °C, followed by washing for 3 min at the same shear rate with modified Tyrodes buffer whilst fluorescent images were captured. Adherent cells were then fixed with 3.7% paraformaldehyde for 30 min and imaged using differential interference contrast (DIC) microscopy on a Zeiss Axiovert 200 m microscope (Carl Zeiss Ltd, Welwyn Garden City, UK).

### Immunoprecipitation and Western blotting

Washed platelets were pre-treated with 1 mm EGTA, 10 μm indomethacin and 2 U mL^−1^ apyrase to inhibit platelet aggregation, thromboxane A_2_ (TxA_2_) production and to block adenosine 5’-diphosphate (ADP), respectively. Platelets were stimulated with agonists at 37 °C with stirring at 1200 r.p.m. in a Born lumi-aggregometer. Reactions were terminated by addition of 2× ice-cold NP-40 lysis buffer. Platelet lysates were pre-cleared and detergent insoluble debris was discarded [24]. A small aliquot was dissolved with sodium dodecyl sulfate (SDS) sample buffer for detection of total tyrosine phosphorylation. Antibodies against PLCγ2, Syk, SLP-76, LAT, Gads, Grb2 or an isotype control were added to the resultant supernatant and incubated overnight. The proteins were immunoprecipitated by addition of protein A-Sepharose or protein G-Sepharose beads. Precipitated proteins or whole-cell lysates were separated by SDS- polyacrylamide gel electrophoresis, electrotransferred, and Western blotted by the indicated antibodies.

### Platelet spreading assay

Coverslips were coated with matrix proteins as previously reported [26]. Platelets were spread on collagen for 45 min at 37 °C in the presence of apyrase (2 U mL^−1^) and indomethacin (10 μm) before washing with PBS followed by fixation with paraformaldehyde (3.7%). Platelets were imaged by DIC microscopy on a Zeiss Axiovert 200 m microscope. Platelet surface area was analyzed using ImageJ (NIH, Bethesda, USA) [26].

## Results

### Gads is required for rapid onset of aggregation and secretion by low concentrations of CRP

**Fig. 1:**
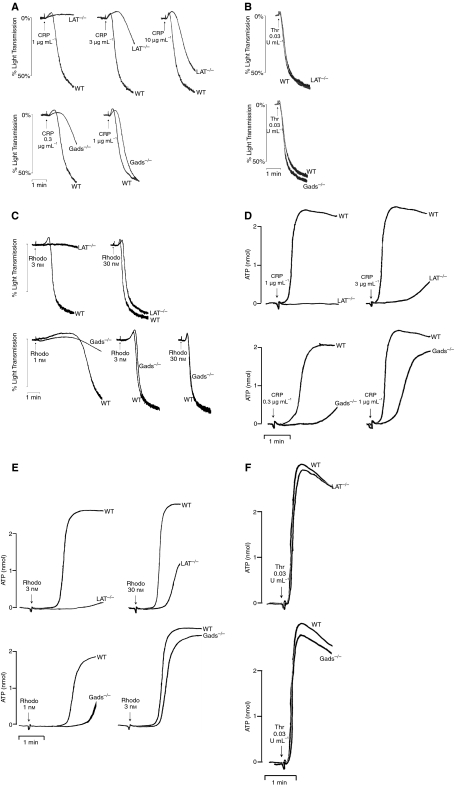
Gads is required for rapid onset of aggregation and secretion by low concentrations of CRP and rhodocytin. Washed mouse platelets (2 × 10^8^ mL^−1^) were stimulated in an aggregometer with CRP (A), thrombin (B) or rhodocytin (C) and allowed to aggregate. Percentage light transmission was calculated. Washed mouse platelets (2 × 10^8^ mL^−1^) were stimulated in an aggregometer with CRP (D), rhodocytin (E) or thrombin (F) and allowed to aggregate for 2 min. Adenosine triphosphate secretion was measured using light emission from luciferin/luciferase. Results are representative of between three and eight experiments.

A role for Gads and LAT in supporting platelet aggregation was investigated using knockout mice. The GPVI receptor agonist, CRP, stimulated concentration-dependent aggregation, with a threshold at 0.1 μg mL^−1^ and a maximal response at 1 μg mL^−1^ (not shown). In the absence of LAT, the dose–response curve was moved approximately tenfold to the right, such that a concentration of 10 μg mL^−1^ of CRP was required to induce full aggregation, although it was notable that this response was delayed in onset (Fig. 1A). In contrast, the dose–response curve for aggregation to CRP was only slightly right-shifted in the absence of Gads, although there was a clear delay in the onset of response to lower concentrations of the synthetic collagen. Similar observations were observed on Gads^−/−^ mice bred on a Balb/c background or on a C57Bl6J background (not shown). In comparison, aggregation induced by a low concentration of the G protein-coupled receptor agonist thrombin (0.03 U mL^−1^) was not significantly different in the absence of LAT or Gads (Fig. 1B).

We investigated the ability of the CLEC-2 receptor agonist, rhodocytin, to stimulate aggregation in the absence of Gads and LAT. We observed a delay and a reduction in aggregation to low concentrations of rhodocytin (1–3 nm) in the absence of Gads, although this effect was not seen with higher concentrations of the snake toxin (Fig. 1C). Similar results were observed on Gads^−/−^ mice bred on a Balb/c or C57Bl6J background (not shown). In contrast, we observed a much greater reduction in response to rhodocytin (3 nm) in LAT-deficient platelets, which could be overcome by a higher concentration (30 nm), although a small delay in aggregation could still be observed.

To investigate a role for Gads in dense granule secretion, we monitored release of ATP using luciferin–luciferase. This revealed a marked delay in the onset of secretion to CRP (0.3–1 μg mL^−1^) in the absence of Gads, although full recovery of response was seen over time (Fig. 1D). A similar pattern of inhibition was observed for expression of the α-granule marker, P-selectin (not shown). In comparison, secretion of ATP induced by CRP (1 μg mL^−1^) was abolished in LAT-deficient platelets, and markedly delayed at the higher concentration of 3 μg mL^−1^. There was also a reduction and delay in ATP secretion induced by a low concentration of rhodocytin (1 nm) in Gads-deficient platelets, although near full recovery was seen at a higher concentration (Fig. 1E). In comparison, the response to 3 nm rhodocytin was abolished in the absence of LAT, with partial recovery observed in response to a higher concentration (30 nm). The responses to low concentrations of thrombin were not altered in the absence of Gads or LAT (Fig. 1F).

Thus, the above observations demonstrate that Gads plays a relatively minor role in mediating platelet activation by GPVI and CLEC-2 in contrast to the greater role of LAT.

### Spreading of Gads-deficient platelets on other matrix proteins

**Fig. 2:**
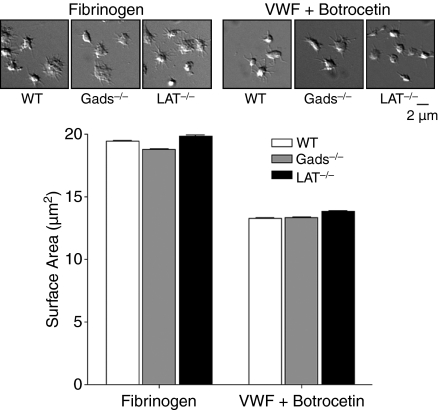
Spreading of Gads-deficient and LAT-deficient platelets to other matrix proteins. Washed mouse platelets (2 × 10^7^ mL^−1^) were allowed to spread on fibrinogen or botrocetin treated von Willebrand factor coated cover-slips for 45 min at 37 °C. Non-adherent platelets were subsequently washed away and adherent platelets were fixed and imaged by differential interference contrast (DIC) microscopy. Surface area of adherent platelets was calculated using ImageJ software. The results are representative of five fields of view from three mice ± 95% confidence interval. Statistical significance was calculated using a Student’s *t*-test.

The above studies were extended to investigate the role of Gads and LAT in spreading of platelets on fibrinogen and von Willebrand factor (VWF)/botrocetin, which is mediated through activation of Syk downstream of integrin α_IIb_β_3_ and GPIb–IX–V, respectively [26,27]. Platelets that have been allowed to spread on fibrinogen form filopodia and limited lamellipodia, whereas platelets spread on VWF/botrocetin form filopodia only. As shown in Fig. 2, there was no significant difference in the surface area or morphology of control and Gads^−/−^ and LAT^−/−^ platelets that had been allowed to spread on fibrinogen or VWF/botrocetin.

### Platelet aggregation on collagen at arteriolar rates of flow

**Fig. 3:**
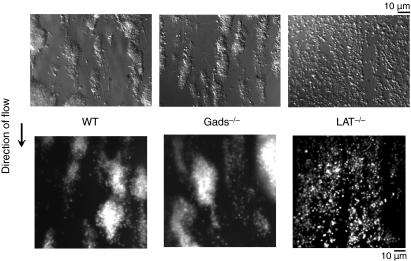
Adhesion of Gads-deficient platelets on collagen under flow conditions. Heparinized whole mouse blood was passed through collagen-coated glass capillaries at an intermediate shear rate of 1000 s^−1^. Platelets were fixed and subsequently imaged by differential interference contrast (DIC) microscopy (upper). Platelets were labeled fluorescently with DiOC_6_ and imaged with a fluorescent microscope (lower). Results are representative of five experiments.

Studies were designed to monitor platelet aggregation on collagen at an arteriolar rate of flow in platelets deficient in Gads or LAT. There was no detectable difference in the time course or magnitude of platelet aggregation on collagen in the absence of Gads relative to control platelets (Fig. 3). In comparison, LAT^−/−^ platelets formed a monolayer on collagen but were unable to form platelet aggregates. This can be explained by the limited degree of platelet activation giving rise to stable adhesion, whereas secretion is required for subsequent recruitment and activation of platelets.

### Measurement of protein tyrosine phosphorylation in Gads-deficient platelets

**Fig. 4:**
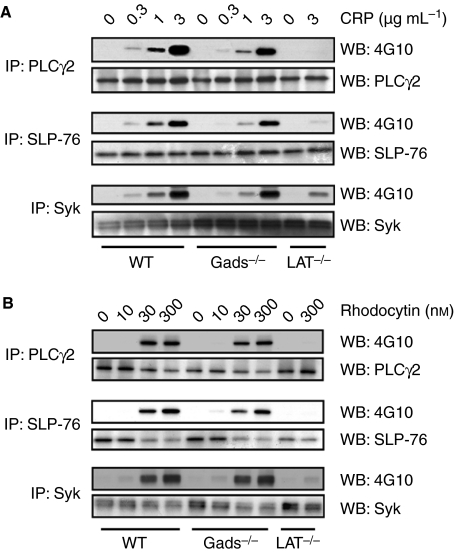
Measurement of protein tyrosine phosphorylation in Gads-deficient platelets. Washed mouse platelets (5 × 10^8^ mL^−1^) were stimulated with CRP for 60 s (A) or rhodocytin for 120 s (B) and subsequently lysed with NP-40 detergent. PLCγ2, SLP-76, and Syk were immunoprecipitated using pAbs and proteins were separated by reducing sodium dodecyl sulfate polyacrylamide gel electrophoresis (10%) and transferred to polyvinylidene fluoride membrane. The membrane was probed with an anti-pTyr mAb, and reprobed with PLCγ2, SLP-76 or Syk pAbs. Results are representative of between four and six experiments.

The above results reveal a minor role for Gads in supporting platelet aggregation and secretion downstream of GPVI and CLEC-2. To investigate the molecular basis of this, we measured tyrosine phosphorylation of the key signaling proteins, Syk, SLP-76, and PLCγ2. There was a small but consistent reduction in tyrosine phosphorylation of Syk, SLP-76, and PLCγ2 induced by CRP in the absence of Gads (Fig. 4A), consistent with a supporting role for Gads in mediating activation of PLCγ2. In comparison, there was a marked inhibition of phosphorylation of all three proteins in the absence of LAT in response to CRP, as previously reported [12]. There was also a minor reduction in phosphorylation of SLP-76 and PLCγ2 in Gads^−/−^ platelets in response to rhodocytin, although phosphorylation of Syk was not altered (Fig. 4B). Phosphorylation of all three proteins induced by rhodocytin was inhibited in the absence of LAT. These results are in line with those for aggregation and secretion, with minor and major roles for Gads and LAT, respectively, in regulating phosphorylation of PLCγ2 downstream of GPVI and CLEC-2.

### Immunoprecipitation of Gads and Grb2

**Fig. 5:**
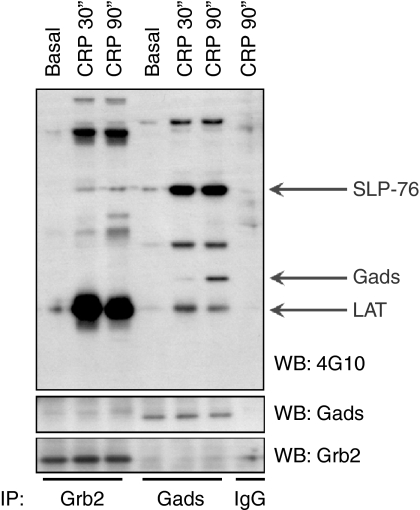
Gads associates with LAT and SLP-76 in platelets. Washed platelets (5 × 10^8^ mL^−1^) were stimulated with 10 μg mL^−1^ CRP for 30 and 90 s and subsequently lysed with NP-40 detergent. Gads and Grb2 were immunoprecipitated using pAbs and proteins were separated by reducing sodium dodecyl sulfate polyacrylamide gel electrophoresis (4–12%) and transferred to polyvinylidene fluoride membrane. The membrane was probed with an anti-pTyr mAb, and reprobed with the Gads and Grb2 pAbs. Results are representative of five experiments.

The relatively minor role of Gads in mediating platelet activation by CRP and rhodocytin raises the issue of whether there is a Gads-related protein that supports platelet activation downstream of GPVI and CLEC-2 through binding to LAT. Gads belongs to a family of three adapters, but only one other of these is expressed in platelets, namely Grb2, which is also known to bind to LAT [28]. To compare the roles of Gads and Grb2, both proteins were immunoprecipitated from basal and CRP-stimulated platelets and samples analyzed for protein tyrosine phosphorylation (Fig. 5). Tyrosine phosphorylated bands of 38 and 76 kDa, which co-migrate with LAT and SLP-76, respectively, were observed to immunoprecipitate with Gads, along with a band of 45 kDa that was detected after 90 s, and which co-migrates with a band that has previously been identified as Gads [17]. Two further, unidentified tyrosine phosphorylated bands of 60 and 150 kDa were also present. In comparison, a major tyrosine phosphorylated band of 38 kDa that co-migrates with LAT is observed in the Grb2 immunoprecipitates, along with a weakly tyrosine phosphorylated band of 76 kDa that co-migrates with SLP-76. There is also a prominent tyrosine phosphorylated band of 125 kDa that has not been identified. Confirmation that the 38 and 76 kDa bands correspond to LAT and SLP-76 was achieved by immunoprecipitation of both proteins and Western blotting (not shown).

## Discussion

The present study was undertaken with the primary goal of comparing the roles of Gads and LAT in the regulation of PLCγ2 by platelet glycoprotein receptors, with particular emphasis on the collagen receptor GPVI and the C-type lectin receptor CLEC-2. In agreement with previous studies, LAT was demonstrated to be critical for GPVI-mediated aggregation and secretion, with only high concentrations of CRP inducing aggregation in the LAT^−/−^ mice. Further, LAT^−/−^ platelets form a monolayer but are unable to aggregate when flowed over collagen at an arteriolar rate of flow, consistent with the critical role for dense granule secretion in mediating aggregate development [29,30]. LAT is also critical for secretion and aggregation induced by low concentrations of the CLEC-2 ligand rhodocytin, whereas recovery is seen at higher concentrations. However, LAT does not play a role in signaling by integrin α_IIb_β_3_ or GPIB–IX–V. In comparison, Gads has a minor role in mediating platelet activation by GPVI and CLEC-2, as illustrated by the delay in onset and reduction in aggregation and secretion following activation by low but not high concentrations of CRP and rhodocytin, respectively. Further, Gads does not mediate spreading by integrin α_IIb_β_3_ and GPIb–IX–V, although this is not surprising, given the lack of a role for LAT in this event. Thus, these results illustrate a differential role for LAT and Gads in supporting platelet activation by GPVI and CLEC-2, whereas neither plays a role in mediating spreading induced by integrin α_IIb_β_3_ and GPIb–IX–V.

The minor role for Gads in supporting GPVI signaling in platelets is in line with its more limited role in TCR signaling relative to that of LAT and SLP-76. For example, mice deficient in Gads have a marked reduction in mature T cells [9] as a consequence of inhibited pre-TCR signaling, whereas mice deficient in LAT and SLP-76 have no circulating T cells. Further, mutation of the site of interaction of Gads with LAT reduces signaling through the T-cell receptor by approximately 50% [28] in contrast to the full inhibition of T-cell signaling that is observed in the absence of LAT and SLP-76 [7,8]. Thus, Gads plays a relatively minor role in both platelets and T cells in comparison with that of LAT and SLP-76, and its major function may be to facilitate the interaction of the two adapter proteins in response to low, threshold levels of receptor stimulation. In theory, the breeding of mice that are deficient in both Gads and LAT is required to confirm a role for Gads in the regulation of LAT in that a similar level of inhibition should be seen in mice platelets deficient in LAT to those deficient in LAT and Gads. However, the relatively small phenotype observed in the absence of Gads would render it difficult to obtain an unequivocal answer on this.

Platelets express the Gads-related adapter, Grb2, which also binds to LAT and SLP-76. This raises the possibility that a role for Gads could be masked by the presence of Grb2, especially in light of studies in a DT40 cell model reporting that Gads and Grb2 are both able to couple LAT and SLP-76, albeit that Gads does this more efficiently [31]. Indeed, this is consistent with the present result, which demonstrates that Grb2 associates strongly with LAT but only weakly with SLP-76, whereas Gads is constitutively associated with SLP-76 (not shown). The strong binding of Grb2 to LAT is explained by the presence of three sites for association of Grb2 with LAT at phosphotyrosines Y171, Y191, and Y226 [28,32,33], compared with a single site for Gads at phosphotyrosine Y191, and to a lesser extent Y171 [28,32,33]. However, the increased binding of Gads to SLP-76 is explained by the very high affinity of the association between the C-terminal SH3 domain of Gads and a RxxK motif on SLP-76 of 3 nm [34,35]. This is an atypical interaction, as the majority of SH3 domains bind to proline rich sequences with micromolar affinity, including the C-terminal SH3 domain of Grb2, which binds to a proline rich region in SLP-76 with an affinity of 3 μm [34].

Overall, the present observations and previous data emphasize that SLP-76 and LAT are the key adapters in bringing PLCγ2 to the membrane, with SLP-76 being essential for tyrosine phosphorylation and activation of the phospholipase. In the absence of LAT, a limited degree of tyrosine phosphorylation activation of PLCγ occurs, which is sufficient to enable recovery of aggregation to high concentrations of CRP in the absence of shear. However, platelet aggregation on collagen at arteriolar shear is abolished in the absence of LAT (present results) resulting in increased tail-bleeding and impaired thrombus formation *in vivo* [36]. Interestingly, a reduction in tyrosine phosphorylation of Syk was also observed in the absence of LAT in platelets stimulated by CRP and rhodocytin. This reduction is most likely due to increased accessibility of tyrosine residues in Syk to protein tyrosine phosphatases in the absence of LAT. In addition, however, we have also identified a novel positive feedback role for Syk in mediating its own phosphorylation in rhodocytin-stimulated platelets, which also contributes to this reduction in phosphorylation (Spalton JC, Watson SP, unpublished).

In comparison to LAT and SLP-76, Gads has a relatively minor role in recruiting SLP-76 to LAT and activation of PLCγ2, and does not contribute to platelet aggregation at arteriolar shear. Indeed, it may be that the evolution of a role for Gads in mediating the interaction between LAT and SLP-76 has occurred because of its role in facilitating weak signaling by the pre-TCR to ensure an optimal number of mature T cells in the circulation rather than to facilitate TCR or platelet activation at higher agonist concentrations. Consistent with this theory, Gads can be bypassed in an active PLCγ signalosome as PLCγ can be recruited to LAT through a direct interaction between LAT Y132 and its N-terminal SH2 domain [37–39].

The differing dependency on LAT and SLP-76 in the GPVI and CLEC-2 signaling cascades could reflect either the presence of a LAT-like molecule in platelets or the presence of a LAT-independent pathway of recruitment of SLP-76 to the membrane. The present study does not distinguish between these two possibilities. However, in a related line of work in the laboratory, we have been unable to find evidence for a functional role of the known LAT-like adapters in mediating platelet activation by GPVI (Pearce AC, Schraven B, Hughes CE, Watson SP, unpublished data). Thus, we favor a model in which SLP-76 and PLCγ2 are able to interact at the membrane independent of a LAT-like membrane adapter protein, with Gads and LAT having evolved to increase the magnitude of signaling. Thus, the mechanism of activation of LAT-deficient platelets by GPVI and CLEC-2 may be similar to that used by integrin α_IIb_β_3_ and GPIb–IX–V, which induce relatively weak activation through LAT-independent, SLP-76-dependent activation of PLCγ2.

In conclusion, the present study has demonstrated that the adapter Gads plays a minor role in mediating platelet activation by GPVI and CLEC-2, emphasizing an alternate pathway through which LAT and SLP-76 regulate PLCγ2.
